# Draft Genome Assembly and Transcriptome Dataset for European Turnip (*Brassica rapa* L. ssp. *rapifera*), ECD4 Carrying Clubroot Resistance

**DOI:** 10.3389/fgene.2021.651298

**Published:** 2021-07-02

**Authors:** Sin-Gi Park, Eonji Noh, SuRyun Choi, Boram Choi, In-Gang Shin, Seung-il Yoo, Dong Jin Lee, Sumin Ji, Hae-Suk Kim, Yoon-Jung Hwang, Jung Sun Kim, Jacqueline Batley, Yong Pyo Lim, David Edwards, Chang Pyo Hong

**Affiliations:** ^1^Theragen Bio Co., Ltd., Suwon, South Korea; ^2^Department of Horticulture, College of Agriculture and Life Science, Chungnam National University, Daejeon, South Korea; ^3^Department of Chemistry Life Science, Sahmyook University, Seoul, South Korea; ^4^Genomics Division, National Institute of Agricultural Sciences, Rural Development Administration, Jeonju, South Korea; ^5^School of Biological Sciences and Institute of Agriculture, University of Western Australia, Perth, WA, Australia

**Keywords:** *Brassica rapa* L. ssp. *rapifera*, clubroot resistance, draft genome, European turnip, genome assembly, orthologous gene cluster, *R* gene

## Introduction

The *Brassica* genus consists of economically important oil and leafy vegetable crops, which are cultivated worldwide. The *Brassica* species represent as the “U's triangle” (Nagahara, 1935), which includes the three basic diploid species *Brassica rapa* (A genome), *Brassica nigra* (B genome), and *Brassica oleracea* (C genome), as well as the three amphidiploid species *Brassica juncea* (A and B genomes), *Brassica napus* (A and C genomes), and *Brassica carinata* (B and C genomes). *Brassica rapa* (2*n* = 2*x* = 20) has been cultivated for specific phenotypic characteristics such as heading (i.e., ssp. *pekinensis*; Chinese cabbage) and non-heading (i.e., ssp. *chinesis*; pak choi) leafy vegetables, tuberized hypocotyl/roots (i.e., ssp. *rapifera*; turnip), and oil rich seedpods (i.e., ssp. trilocularis; yellow sarson) in the oil crops. Although the genomes of its subspecies are very similar (Lin et al., 2014), *B. rapa* demonstrates extreme morphological diversity. Because of the economical value and scientific interest in phenotypic diversity, the genome sequence of mesopolyploid *B. rapa* ssp. *pekinensis* Chiifu, a Chinese cabbage, was the first published *B. rapa* reference genome, and revealed that *B. rapa* evolved via a two-step whole-genome triplication and, as a result, has three syntenic subgenomes (Wang et al., 2011). The polyploidization is hypothesized to have facilitated the diversification of genes as well as gene fractionation, and as a consequence led to the evolution of different morphotypes within and between related *Brassica* species. The study of intraspecific diversity in *B. rapa* offers opportunities to advance our understanding of plant growth, development, and phenotypic evolution (Paterson et al., 2001).

Over the last few years, the genome assembly of *B. rapa* (version 3.0) has been improved using single-molecule sequencing, optical mapping, and chromosome conformation capture technologies (Hi-C), resulting in an approximately 30-fold improvement with a contig N50 size of 1.45 Mb compared with that of previous references (Zhang et al., 2018). The assembly refined the syntenic relationship of genome blocks and centromere locations in the genome, and identified a greater number of annotated transposable elements (TEs) than in previous assemblies. In addition to the whole-genome sequence of Chinese cabbage, chromosome-level genome sequences of other *B. rapa* subspecies, including yellow sarson (Belser et al., 2018) and pak choi (Li P. et al., 2020; Li Y. et al., 2020), have been recently reported. The studies provide insight to the understanding of genetic drivers underlying the morphological variation among *B. rapa* subspecies. Moreover, a pangenome from Chinese cabbage, rapid-cycling *Brassica*, and Japanese vegetable turnip was constructed using Illumina short-read data (Lin et al., 2014), identifying genomic determinants of morphological variation, especially copy number differences in peroxidases associated with the phenylpropanoid biosynthetic pathway in turnip.

Turnip (*B. rapa* L. ssp. *rapifera*) represent one of the morphotypes in *B. rapa* that forms tubers (hypocotyl/taproot tubers), produces lobed leaves with long petioles, and can be used to study the genetics underlying storage organ formation (Zhang et al., 2014). Brassica species are susceptible to clubroot disease, caused by *Plasmodiophora brassicae* (Schwelm et al., 2015), and forms galls (clubs) on infected root tissues with abnormal proliferation, preventing water and nutrient uptake and retarding the normal growth and development of plants, resulting in significantly reduced yield and quality. Sources of known clubroot resistance genes are derived from European turnip, carrying strong resistance to this disease (Matsumoto et al., 1998; Hirani et al., 2018). The resistance genes of European turnip have been introduced into other *Brassica* crops including vegetables and oilseed rape. In particular, European clubroot differential (ECD) turnips, that exhibited high levels of resistance to clubroot and that consist of four accessions (ECD1–ECD4), were developed and have been helpful for discovering dominant loci conferring clubroot resistance genes including *CRa, CRb*, and *CRc* for marker-assisted selection in canola and other *Brassica* species (Hirani et al., 2018).

Here, we report the draft genome assembly of a European turnip, ECD4, which has strong clubroot resistance, that was generated by PacBio single-molecule long-read sequencing technology. This draft genome assembly coupled with transcriptome data derived from various leaf and root tissues will support the discovery of disease resistance genes (*R* genes) especially clubroot resistance genes, enabling the development of allele-specific markers for marker-assisted selection in *Brassica* breeding. Moreover, these data provide a valuable resource for studying the morphological diversity and evolution of turnips.

## Data Briefs

### Whole-Genome *de novo* Assembly of European Turnip

**Figure 1:**
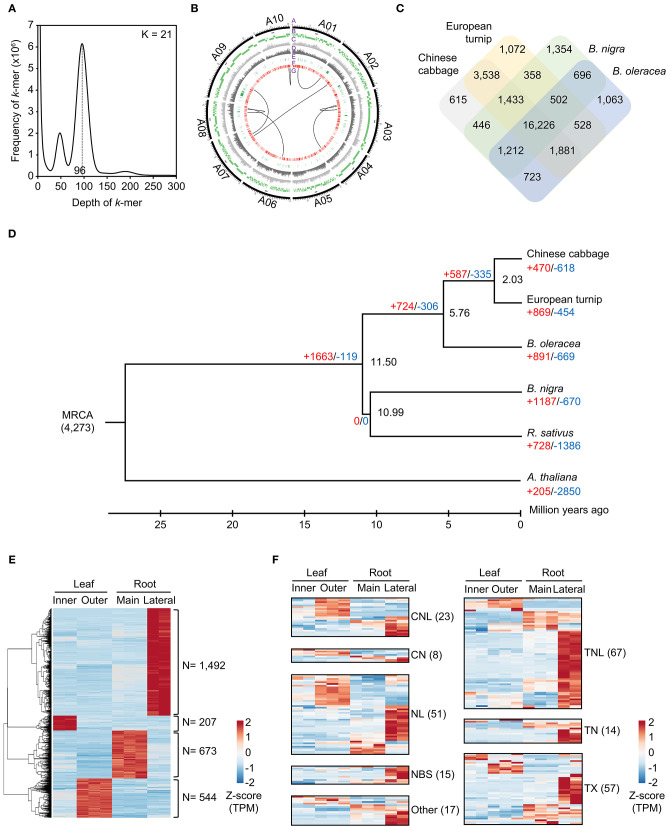
Characterization of the draft genome assembly for the European turnip ECD4. **(A)** Estimation of genome size on the basis of *k*-mer frequency analysis. **(B)** Anchoring genome assembly data of European turnip to 10 chromosomes of Chinese cabbage (A1–A10). In the Circos plot, A, B, C, D, E, F, and G indicate chromosomes of Chinese cabbage, alignment of genome assembly data of European turnip, SNPs, InDels, NBS-encoding *R* genes, membrane-associated *R* genes, and translocations, respectively. **(C)** Orthologous gene clusters among Chinese cabbage, European turnip, *B. nigra*, and *B. oleracea*. **(D)** Expansion and contraction of gene families based on a time-calibrated phylogeny of four *Brassica* species, *R. sativus*, and the outgroup *A. thaliana*. **(E)** Identification of genes showing tissue-specific expression in the inner and outer leaves and the main and lateral root tissues of European turnip. **(F)** Genome-wide expression of NBS-encoding *R* genes identified in the leaf and root tissues of European turnip.

For the genome assembly of European turnip (ECD4), a total of 70.63 Gb of PacBio long reads (an average sequencing coverage of 136.36x) and 72.16 Gb of Illumina short reads (139.3x) were generated (Supplementary Table 1). Based on *k*-mer analysis (*k* = 21), the estimated genome size of European turnip is approximately 518 Mbp (Figure 1A). The genome assembly resulted in a 315.8 Mb draft genome with 655 contigs (contig N50 length of 1.45 Mb; the longest contig length was 21.92 Mb) (Table 1). Our assembly data covered about 61% of the genome of European turnip, being predicted as mostly euchromatins. The assembly coverage is slightly low compared with those reported in Chinese cabbage (353 Mb) (Zhang et al., 2018), pak choi (370 Mb) (Li P. et al., 2020; Li Y. et al., 2020), and yellow sarson (402 Mb) (Belser et al., 2018). Whole-genome sequences of Chinese cabbage, pak choi, and yellow sarson were assembled at the chromosome-level by using chromosome conformation capture (Hi-C) technology [Chinese cabbage (reference genome version 3.0) and pak choi] and/or BioNano optical mapping [Chinese cabbage (v3.0) and yellow sarson] besides long-read sequencing such as PacBio SMRT sequencing or Oxford Nanopore sequencing. Therefore, such long-range scaffolding technologies are further required to improve the quality of genome assembly in European turnip. While the remained 39% is expected to contain considerably repetitive DNAs, including tandem satellite repeats, rDNAs, and retrotransposons which can disturb assembly (Wang et al., 2011; Perumal et al., 2017; Zhang et al., 2018; Li P. et al., 2020). Of the contigs, 260 were anchored to 10 chromosomes of Chinese cabbage (cv. Chiifu-401) (version 3.0), with a total length of 260.47 Mb (Figure 1B; Supplementary Table 2).

**Table 1:** Genome assembly and gene prediction of European turnip.

**Genome assembly**	
No. of contigs	655
Total length (Mb)	315.77
Contig N50 length (Mb)	1.45
Longest contig length (Mb)	21.92
GC content (%)	35.58%
**Gene prediction**	
No. of genes	48,349
Average length of gene (bp)	1,907
Exon	
No. of exons	266,261
Average number of exons per gene	5.23
Average exon length (bp)	215
Intron	
No. of introns	215,413
Average number of introns per gene	4.23
Average intron length (bp)	183
**Homology-based functional annotation**	45,622 (94.36%)
UniProt/SwissProt	31,538 (65.23%)
TAIR	40,220 (83.19%)
NCBI NR	44,110 (91.23%)
InterPro domains	43,417 (89.80%)

The quality of the genome assembly was assessed using BUSCO and paired-end read mapping. First, the draft genome assembly captured 99.6% of the complete Benchmarking Universal Single-Copy Orthologs (BUSCOs) with the Virdiplantae_odb10 database; 82.4, 17.2, 0.2, and 0.2% of the BUSCOs were predicted as complete and single-copy, complete and duplicated, fragmented, and missing, respectively. The high completeness of our assembly data seems to be resulted from error correction and curation of heterozygous assemblies by Pilon and Purge_haplotigs with high read depth and coverage (read depth: 139.3x of short reads for Pilon and 136.4x of long reads for Purge_haplotigs). In particular, the method led to a saturation of BUSCO scores with one round of error correction in Pilon (Supplementary Table 10). The genome completeness of European turnip is similar to that of Chinese cabbage (Supplementary Table 3). Second, 94.58% of the short-insert paired-end reads were successfully realigned to the assembly, with 99.84% assembly coverage. Moreover, the analysis showed that 0.32% of the nucleotides in the genome assembly were heterozygous.

### Identification of Genome Rearrangements Between European Turnip and Chinese Cabbage

The whole-genome comparison revealed eight genomic rearrangements between European turnip and Chinese cabbage (“G” layer in Figure 1B), with the identification of hotspots (Supplementary Table 4; Supplementary Figure 1), indicating genomic change since the divergence from a common ancestor of *B. rapa*. For example, the positions 629.5 to 774.9 kb (145.4 kb in length) and positions 11.5 to 579.4 kb (567.9 kb in length) on contig00007136 of European turnip were aligned to positions 18,057.7 to 18,217.2 kb (159.5 kb in length) and positions 18,966.7 to 19,568.7 kb (602 kb in length) on chromosome A10 of Chinese cabbage, respectively. These hotspots were validated by mapping Illumina paired-end reads of European turnip to the corresponding hotspot of Chinese cabbage and then identifying the split of paired-end read mapping in Chinese cabbage (Supplementary Figure 8). We further identified five and three genome rearrangements between pak choi and European turnip, and between yellow sarson and European turnip, respectively, showing large genome variations among *B. rapa* subspecies (Supplementary Figure 7). Additionally, 3,795,367 SNPs, with a transition/transversion ratio (Ts/Tv) of 1.35, and 954,051 InDels were identified (“C” and “D” layers in Figure 1B).

### Genome Annotation: Identification of TEs and Gene Prediction and Annotation

A total of 112.6 Mb of TEs were identified, accounting for 35.66% of the genome assembly (Supplementary Table 5). Of the class I elements, *Ty3/Gypsy* (29.5%) and *Ty1/Copia* (18.7%) for long terminal repeat (LTR) retrotransposons and LINE (L1) for non-LTR retrotransposons were abundant in the genome. The *hAT* family (11.7%) represented the most abundant DNA transposon (Supplementary Table 5). A total of 48,349 non-redundant protein-coding genes were predicted with an average number of exons per gene of 5.23 using evidence-driven gene prediction methods coupled with *ab initio* prediction (Table 1). The gene models were supported by 93.04% RNA-Seq data derived from two leaves and two root tissues of European turnip (Supplementary Table 6). Of the predicted genes, 45,622 (94.36%) were successfully annotated by at least one database, including UniProt/SwissProt, TAIR, NCBI NR, and InterPro. To investigate species-specific and shared genes in European turnip compared with other *Brassica* diploids, including Chinese cabbage (AA), *B. nigra* (BB), and *B. oleracea* (CC), we analyzed orthologous gene clusters. This analysis showed that 24,466 of 31,647 orthologous gene clusters were shared among European turnip and three *Brassica* diploids and that 1,072 orthologous gene clusters were species-specific in European turnip (Figure 1C). This highlighted the enrichment of genes with F-box domains that play various roles in developmental processes, including plant hormone signal transduction, floral development, secondary metabolism, senescence, circadian rhythm, and response to biotic and abiotic stress (Xu et al., 2009) (Supplementary Figure 2). Of the orthologous gene clusters, five *R* genes (three TIR- and two CC-NBS-LRR-containing domain proteins) were found. We also identified 709, 672, and 463 orthologous gene clusters specific to European turnip, yellow sarson, and Chinese cabbage, respectively (Supplementary Figure 9A). Similar to above finding, the enrichment of genes with F-box and leucine-rich repeat (LRR) domains was identified in orthologous gene clusters specific to European turnip (Supplementary Figure 9B), reflecting the characteristics of plant disease resistance in European turnip.

### Gene Family Expansions and Contractions

A phylogenetic tree of European turnip was constructed using a total of 3,139 single-copy orthologous genes (Figure 1D). Through orthologous gene cluster analysis, we identified that European turnip has 869 expanded and 454 contracted gene families (red- and blue-colored numbers in Figure 1D). Of the expanded gene families, protein domains found in genes associated with plant development (no apical meristem-associated C-terminal) and plant defense (haloacid dehalogenase-like hydrolase, xylanase inhibitor C-terminal, tetratricopeptide repeat, and oxidative-stress-responsive kinase 1 C-terminal) were enriched (Supplementary Figure 3).

### Tissue-Specific Gene Expression in European Turnip

Tissue-specific gene expression was identified in inner leaf (207 genes), outer leaf (544 genes), main root (673 genes), and lateral root (1,492 genes) tissues, with a statistical cut-off of *q* < 0.05, absolute 2-fold change, and at least one tissue with ≥10 FPKMs (Figure 1E). Functional annotation for these genes revealed the functions for each of tissues; for example, chloroplast organization (*P* = 1.1 × 10^−12^) for inner leaves, phosphorylation (*P* = 1.2 × 10^−23^), and defense response (*P* = 7.1 × 10^−6^) for outer leaves, cell wall organization (*P* = 6.7 × 10^−9^), response to salicylic acid (*P* = 1.7 × 10^−5^), and post-embryonic morphogenesis (*P* = 5.6 × 10^−5^) for the main root, and transcription (*P* = 2.2 × 10^−19^), response to salicylic acid (*P* = 3.9 × 10^−8^), wounding (*P* = 5.8 × 10^−8^), toxic substance (*P* = 4.4 × 10^−7^), abscisic acid (*P* = 5.7 × 10^−8^), and jasmonic acid (2.2 × 10^−6^) for lateral roots were significantly represented (Supplementary Figure 4).

### Identification of Disease Resistance Genes (*R* genes) in European Turnip

We also found a total of 252 plant defense-related *R* genes: 23 CC-NBS-LRRs (CNLs), 8 CC-NBSs (CNs), 67 TIR-NBS-LRRs (TNLs), 14 TIR-NBSs (TNs), 51 NBS-LRRs (NLs), 15 NBSs, 57 TIR-others (TXs), and 17 others (Supplementary Table 7). Interestingly, 103 *R* genes exhibited high expression in lateral root tissue (Figure 1F). In addition to these *R* genes, a total of 1,135 genes−119 receptor-like proteins (RLPs), 742 receptor-like kinases (RLKs), and 274 TM-CCs—categorized into membrane-associated *R* genes were identified (Figure 1F; Supplementary Table 7). Of those three gene families, 243 RLKs also showed high expression in the lateral roots (Supplementary Figure 5). Additionally, two contigs harboring microsatellite markers linked to clubroot resistance loci *BraA.CR.a* and *BraA.CR.b* (Hirani et al., 2018), which are located on chromosomes A3 and A8, respectively, were found (Supplementary Table 8; Supplementary Figure 6). Interestingly, the comparison of *R* genes between the linked regions of the two species showed the expansion of *R* genes, mostly *R* genes of TM-CC and TNL classes, in European turnip even though exhibiting their co-linearity in the linked regions (Supplementary Table 9). Moreover, three R genes homologous to *Rcr6*, which is a clubroot resistance gene of TNL class identified in *B. nigra* (Chang et al., 2019), were identified in European turnip, but only one was identified in Chinese cabbage (Supplementary Table 9).

### Summary

We report the first draft genome of European turnip (ECD4) coupled with transcriptome data derived from various tissues. Our data can provide an invaluable genomic resource to study the morphological diversity and evolution of European turnip by applying pan-genomics to *Brassica*. Moreover, our data can help develop molecular markers as a genetic breeding tool to identify plant defense-related genes such as clubroot resistance genes (*R* genes).

## Materials and Methods

### Sample Collection, Library Construction, and Sequencing

Seeds of European turnip ECD4 (*B. rapa* ssp. *rapifera*) (accession no. 25026) were obtained from the Korea Brassica Genome Resource Bank (KBGRB, South Korea). The seeds were germinated in seedling trays containing autoclaved soil in a controlled chamber at 25°C, 60% humidity, and photoperiodic lighting (16 h of light:8 h of dark). From leaf tissue of 3-week-old seedlings, genomic DNA was extracted by a WizPrep Plant DNA Mini Kit (Wizbiosolutions, Seongnam, South Korea) according to the manufacturer's protocol for whole-genome sequencing. A long-read library with high-quality genomic DNA of ≥20 μg was prepared using the SMRTbell Express Template prep kit 2.0 (Pacific Biosciences, Menlo Park, CA, USA) and sequenced on a PacBio Sequel System using one SMRT cell. Additionally, DNA sequence libraries were prepared from 1 μg input DNA using a TruSeq Nano DNA Sample Prep Kit according to the manufacturer's instructions (Illumina, Inc., San Diego, CA, USA). The libraries were subjected to paired-end sequencing with a 150-bp read length using the Illumina NovaSeq 6000 platform.

### Whole-Genome Assembly

Illumina short reads were processed by Jellyfish (version 2.2.0) (Marcais and Kingsford, 2011) to estimate the genome size of European turnip. The *k*-mer frequency with a *k*-mer size of 21 was counted and plotted as a *k*-mer frequency distribution. PacBio SMRT long reads were assembled using CANU (version 1.8) (Koren et al., 2017) with the following primary parameters; genomeSize 518M, minReadLength 1000, minOverlapLength 500, rawErrorRate 0.3, correctedErrorRate 0.045. For assembly polishing, short reads were aligned to the CANU assembly data using BWA-MEM (version 0.7.17) (Li and Durbin, 2009), and errors in nucleotides in the CANU assembly data were then corrected using Pilon (version 1.22) (Walker et al., 2014) with one round of polishing. The polished contigs were processed with Purge_haplotigs to curate heterozygous diploid genome assemblies (Roach et al., 2018). After filtering of ≤10 kb of contigs, a total of 655 assembled contigs were finally generated. This method was summarized in Supplementary Figure 10.

The genome completeness of the draft genome assembly of European turnip was assessed by using BUSCO (version 4.0.5) (Seppey et al., 2019) with 425 single-copy orthologs of the viridiplantae_odb10 database. For estimation of sequence coverage, Illumina short-insert paired-end reads were realigned to the draft genome assembly using BWA-MEM. Furthermore, heterozygous nucleotides were identified by using SAMtools mpileup (Li, 2011).

### Genome Annotation

A combination of *ab initio* and evidence-based approaches was employed for gene prediction of European turnip. The assembled genome was premasked for repetitive DNA sequences using RepeatMasker (version 4.0.6) (http://www.repeatmasker.org/). An unsupervised training gene structure was generated using GeneMark-ET (version 4.10) (Lomsadze et al., 2014) by incorporating RNA-Seq data. *De novo* prediction was performed using AUGUSTUS (version 3.3.1) (Keller et al., 2011) with the training gene set and with exon–intron boundary information predicted by RNA-Seq and protein sequence alignments. Here, STAR (version 2.7.1a) (Dobin et al., 2013) was used for RNA-Seq alignment of the species, and GenomeThreader (version 1.7.0) (Gremme et al., 2005) was used for protein sequence alignment with the *B. rapa* protein sequences (version 3.0). Functional annotation for predicted genes was performed on the basis of homology-based searches with TAIR11, UniProt/SwissProt, and NCBI non-redundant (NR) databases using BLASTP (version 2.3.0+) (Altschul et al., 1990) with a cutoff *E*-value of 1*E*-10. Protein domains were also searched by using InterProScan (version 5.19-58.0) (Mulder and Apweiler, 2007). Additionally, tRNA- and miRNA-like sequences were predicted using tRNAscan-SE (version 1.4 alpha) (Schattner et al., 2005) and Infernal (version 1.1.1) (Nawrocki and Eddy, 2013). Resistance gene analogs (RGAs), such as NBS-encoding proteins, RLKs and RLPs, were predicted using an RGAugury pipeline (Li et al., 2016).

### Ortholog and Phylogenetic Analysis

The protein sequences from turnip (AA) and diploid *Brassica* species, including Chinese cabbage (AA; reference genome v3.0), *B. nigra* (BB; v1.1), *B. oleracea* (CC; v1.1), *Raphanus sativus* (v1.1), and the outgroup species *Arabidopsis thaliana* (version TAIR10) were used to predict orthologous gene clusters. The gene set of each species was filtered as follows: First, the genes encoding proteins of fewer than 30 amino acids were filtered out. Second, the similarity relation between the protein sequences of all of the species was obtained through BLASTP (i.e., all-vs.-all BLASTP searches) with a cutoff E-value of 1*E*-05. All of the protein datasets of the representative species were clustered into paralogues and orthologs by OrthoMCL (version 2.0.9) (Li et al., 2003), with an inflation parameter of 1.5.

The single-copy orthologs in these six species were aligned using MAFFT (version 7.123b) (Katoh and Standley, 2013) and then used to extract conserved regions with Gblocks (version 0.91b) (Talavera and Castresana, 2007). The alignment results were combined to create a superalignment matrix. A phylogenetic tree of these six species was constructed using MEGA X (Kumar et al., 2018) with the maximum likelihood method and 1,000 bootstrap values. The divergence time of European turnip was estimated using RelTime of MEGA X.

### Gene Family Expansion and Contraction Analysis

The expansion and contraction of the gene families were analyzed by comparing the cluster size differences between the ancestor and each species by using café (De Bie et al., 2006). A random birth and death model was used to study the changes in gene families along each lineage of the phylogenetic tree. A probabilistic graphical model (PGM) was introduced to calculate the probability of transitions in gene family size from parent to child nodes in the phylogeny. With conditional likelihoods as the test statistics, the corresponding *p*-value in each lineage was calculated, and a *p*-value of 0.05 was used to identify the families that were significantly expanded or contracted. The expanded and contracted genes were then subjected to PFAM functional annotation.

### Transcriptome Analysis

Three-week-old seedlings were transplanted to pots and grown for 4 weeks in a glasshouse. Total RNA from four tissues (the inner leaf, outer leaf, main root, and lateral root) was extracted using an RNeasy Plant Mini Kit (Qiagen, Hilden, Germany). RNA-Seq libraries from 1 μg of purified total RNA were prepared using the TruSeq Stranded mRNA Sample Prep Kit according to the manufacturer's manual (Illumina, Inc., San Diego, CA). cDNA was synthesized and then subjected to an end repair process, addition of a single “A” base, and ligation of the adaptors. Libraries that were purified and enriched with PCR amplification were subjected to paired-end sequencing with a 150 bp read length using an Illumina NovaSeq 6000 platform.

After removal of adaptor sequences and trimming of low-quality sequences (<Q20) using Cutadapt (version 2.8), clean reads were aligned to the draft genome assembly using STAR (version 2.7.1a). Gene expression quantification was performed using RSEM (version 1.3.1) (Li and Dewey, 2011) with TPM values (version 1.3.1). For analysis of tissue-specific expression in the inner/outer leaves and main/lateral roots, differential expression analysis was performed using DESeq2 (version 1.26.0) (Love et al., 2014) with a cutoff of *q* < 0.05 and absolute ≥2-fold change. Functional annotation for genes showing tissue-specific expression was performed by using DAVID (Huang et al., 2007), and relevant gene ontology (GO) terms were selected with a cutoff EASE score <1 × 10^−4^. The expression pattern of the analyzed genes was visualized as a heatmap by using ClustVis (Metsalu and Vilo, 2015) with the correlations and the average linkage method.

### Comparative Genomics

Syntenic blocks between the chromosomes of Chinese cabbage and contigs of European turnip were detected by using SyMAP (version 4.2) (Soderlund et al., 2006) and are shown in a Circos plot. Genome rearrangement events such translocation between the two genomes were verified by using MUMmer (version 3.9.4) (Delcher et al., 2003).

## Data Availability Statement

The datasets presented in this study can be found in online repositories . Sequencing data used in this study are available in the NCBI Sequence Read Archive (SRA) database under the following accession PRJNA690160; JAEOXI000000000 (whole genome assembly), SRR13375884 (PacBio SMRT long read sequencing data), SRR13375883 (Illumina DNA-Seq data), SRR13375896 - SRR13375905 (RNA-Seq data derived from four different tissues). In addition, datasets for functional annotation are available from our website http://turnip.genome.theragenbio.com/BRT_1.0.

## Author Contributions

CH and DE conceived this study. S-GP, BC, I-GS, S-iY, and DL performed the bioinformatics analyses. EN, SJ, H-SK, Y-JH, and JK performed the whole-genome and RNA sequencing. SC and YL prepared the samples and extracted genomic DNA and total RNA. CH, S-GP, JB, and DE wrote the manuscript. All authors read and approved the final manuscript, contributed to the article, and approved the submitted version.

## Conflict of Interest

The authors declare that the research was conducted in the absence of any commercial or financial relationships that could be construed as a potential conflict of interest.
