# Supplementary material for: Draft Genome Assembly and Transcriptome Dataset for European Turnip (Brassica rapa L. ssp. rapifera), ECD4 Carrying Clubroot Resistance
Source: Front Genet. 2021 Jul 2;12:651298. doi: 10.3389/fgene.2021.651298 (PMC8285094; doi:10.3389/fgene.2021.651298)
Supplement: Supplementary file 1 [file Data_Sheet_1.pdf]

Supplementary Material

Supplementary figures

Figure S1. Dot plot analysis between genomes of European turnip and Chinese cabbage. In the dot plot, the *x*- and *y*-axes indicate positions in the contigs of European turnip and the corresponding aligned genomic regions of Chinese cabbage, respectively.

Figure S2. Functional protein domains enriched in the European turnip-specific orthologous gene cluster. Functional protein domains of genes belonging to the European turnip-specific orthologous gene cluster were searched using InterProscan. Enrichment of Pfam domains was analysed by using DAVID with a cutoff EASE score <1*E*-03.

Figure S3. Functional protein domains enriched in expanded gene families within the genome of European turnip. Functional protein domains were identified by a Pfam search, and domain enrichments were analysed by the Z-test (*p*-value <0.001). The heatmap shows the relative abundance (Z-score) of functional domains across six plant genomes: European turnip (*B. rapa* ssp. *rafifera*), Chinese cabbage (*B. rapa* ssp. *pekinensis*), *B. oleracea*, *B. nigra*, *Raphanus sativus*, and *Arabidopsis thaliana*.

Figure S4. Functional enrichment for genes showing tissue-specific expression. RNA-Seq data derived from inner leaf, outer leaf, main root, and lateral root tissues were analysed by using DESeq2 with a cutoff of *q*<0.05, absolute≥2-fold change, and at least one tissue≥10 FPKMs. Functional annotation for genes showing tissue-specific expression was performed by using DAVID with a cutoff EASE score <1*E*-04.

Figure S5. Prediction of membrane-associated resistance genes in the genome of European turnip. RGAs, such as RLKs and receptor-like proteins (RLPs), were predicted using an RGAugury pipeline. A total of 1,135 genes—119 RLPs, 742 RLKs, and 274 TM-CCs—were identified.

Figure S6. Comparison between genomic regions linked to *BraA.CR.a* and *BraA.CR.b* loci in European turnip and their counterparts in Chinese cabbage.

Figure S7. Identification of genome rearrangements between European turnip (ET) and other *B. rapa* subspecies including Chinese cabbage (CC) (v3), pak choi (PC) (Li et al., 2020), and yellow sarson (YS) (Belser et al., 2018). Comparisons: (A) CC vs. ET (presented in Fig. 1B), (B) CC vs. PC, (C) CC vs. YS, (D) PC vs. ET, and (E) YS vs. ET.

Figure S8. Validation of genome rearrangements between European turnip and Chinese cabbage. (A) The strategy for detecting genome rearrangements based on Illumina paired-end read mapping. For example, paired-end reads of European turnip, which were properly mapped to a 600-kb region in its contig00007136, were mapped to 18.243-Mb region for one side and 19.583-Mb regions for other side in A10 of Chinese cabbage, respectively, showing the collapse of paired-end read mapping in the genome of Chinese cabbage. (B and C) The visualization of read mapping in a hotspot using IGV (https://software.broadinstitute.org/software/igv/).

Figure S9. Orthologous gene clusters among *B. rapa* subspecies sequenced (A) and functional annotation for species-specific genes in three subspecies (B). (A) Orthologous gene clusters among Chinese cabbage (v3), European turnip, and yellow sarson (Belser et al., 2018) were analyzed by using OrthoMCL. (B) Functional protein domains of genes belonging to subspecies-specific orthologous gene cluster were searched using InterProscan. Enrichment of InterPro domains was analyzed by using DAVID with the cutoff of EASE score < 1*E*-02.

Figure S10. Procedure of genome assembly for European turnip. In procedure, the yield of generated and filtrated data is summarized for each of steps.

Supplementary tables

Table S1. Summary for whole-genome sequencing data of European turnip

Table S2. Anchoring contigs of European turnip to the genome of Chinese cabbage

Table S3. Assessment of the genome assembly of European turnip using Benchmarking Universal Single-Copy Orthologues (BUSCOs)

Table S4. Identification of genome rearrangements between European turnip and Chinese cabbage

Table S5. Abundance of transposable elements (TEs) identified in the genome of European turnip

Table S6. Summary of RNA-Seq of different tissues of European turnip

Table S7. Identification of plant *R* genes in the genome of European turnip

Table S8. Mapping of microsatellite markers linked to clubroot resistance loci to the draft assembly of European turnip

Table S9. List of *R* genes within conserved blocks that are linked to clubroot resistance loci.

Table S10. BUSCO scores with multiple rounds of error correction in Pilon.

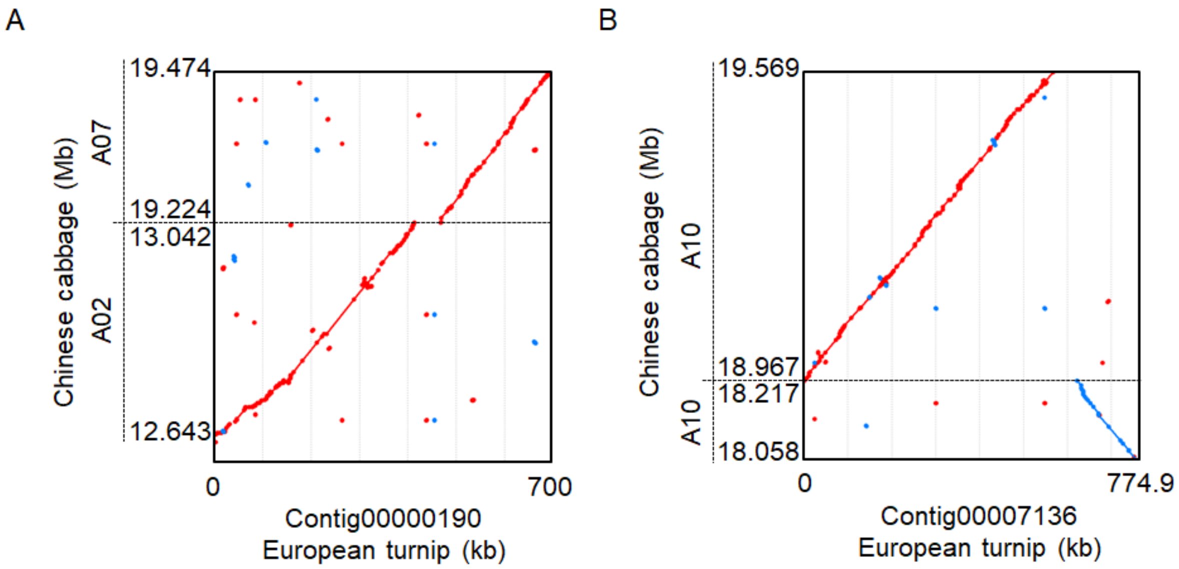

Figure S1. Dot plot analysis between genomes of European turnip and Chinese cabbage. In dot plot, *x*- and *y*-axes indicate positions in contig of European turnip and the corresponding aligned genomic regions of Chinese cabbage, respectively.

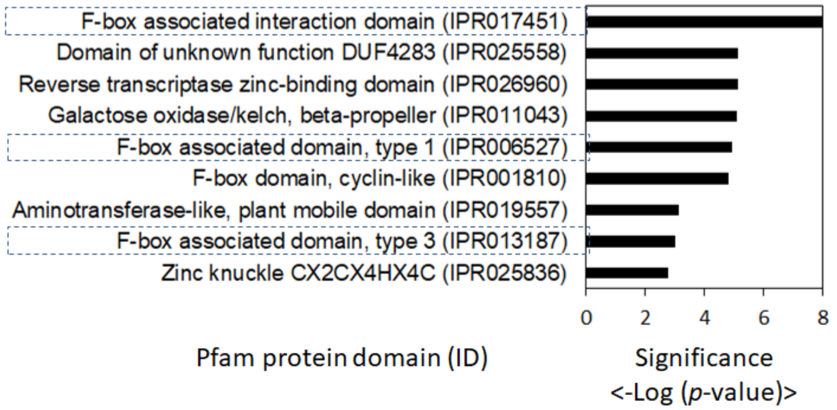

Figure S2. Functional protein domains enriched in European turnip-specific orthologous gene cluster. Functional protein domains of genes belonging to European turnip-specific orthologous gene cluster were searched using InterProscan. Enrichment of Pfam domains was analyzed by using DAVID with the cutoff of EASE score < 1*E*-03.

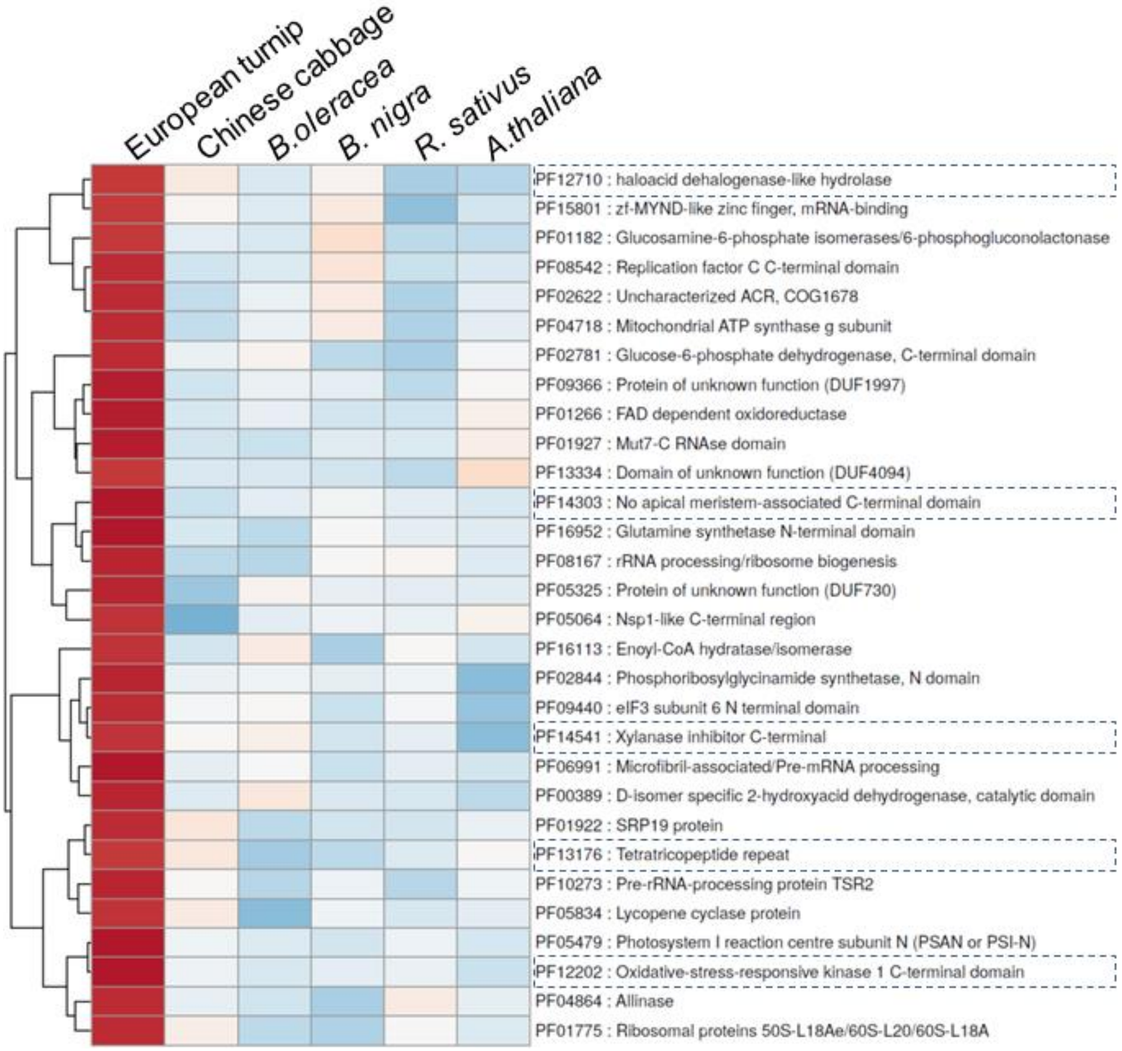

Figure S3. Functional protein domains enriched in expanded gene families within the genome of European turnip. Functional protein domains were identified by Pfam search and domain enrichments were analyzed by the Z-test (p-value < 0.001). The heatmap shows the relative abundance (Z-score) of functional domains across six plant genomes, including European turnip (*B. rapa* ssp. *rafifera*), Chinese cabbage (*B. rapa* ssp. *pekinensis*), *B. oleracea*, *B. nigra*, *Raphanus sativus*, and *Arabidopsis thaliana*.

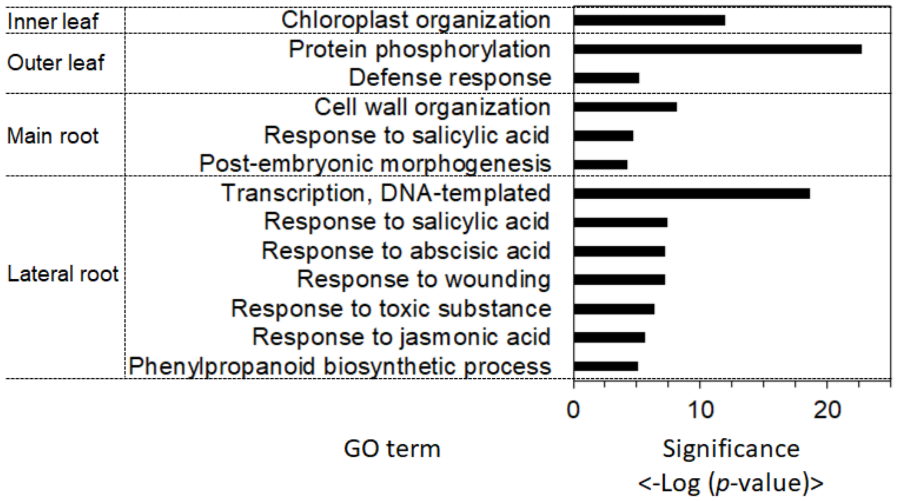

Figure S4. Functional enrichment for genes showing tissue-specific expressions. RNA-Seq data derived from inner leaf, outer leaf, main root, and later root tissues were analyzed by using DESeq2 with the cutoff of $q < 0.05$, absolute ≥ 2-fold change, and at least one tissue ≥ 10 FPKMs. Functional annotation for genes showing tissue-specific expressions was performed by using DAVID with the cutoff of EASE score < 1*E*-04.

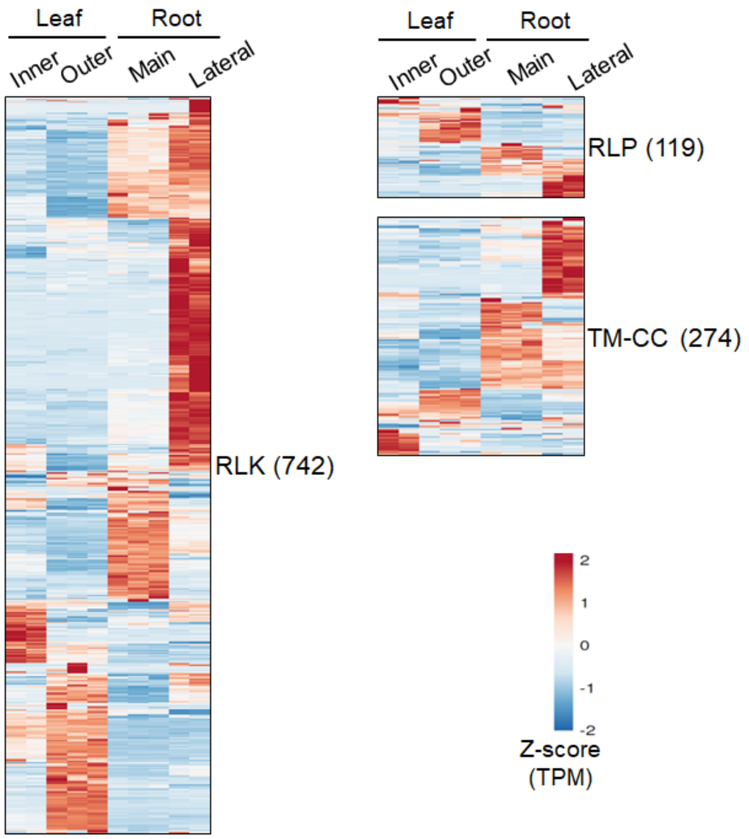

Figure S5. Prediction of membrane-associated resistance genes in the genome of European turnip. The resistance gene analogs (RGAs), such as receptor-like protein kinases (RLKs) and receptor-like proteins (RLPs), were predicted using a RGAugury pipeline. A total of 1,135 genes, including 119 receptor-like proteins (RLPs), 742 receptor-like kinases (RLKs), and 274 TM-CCs, were identified.

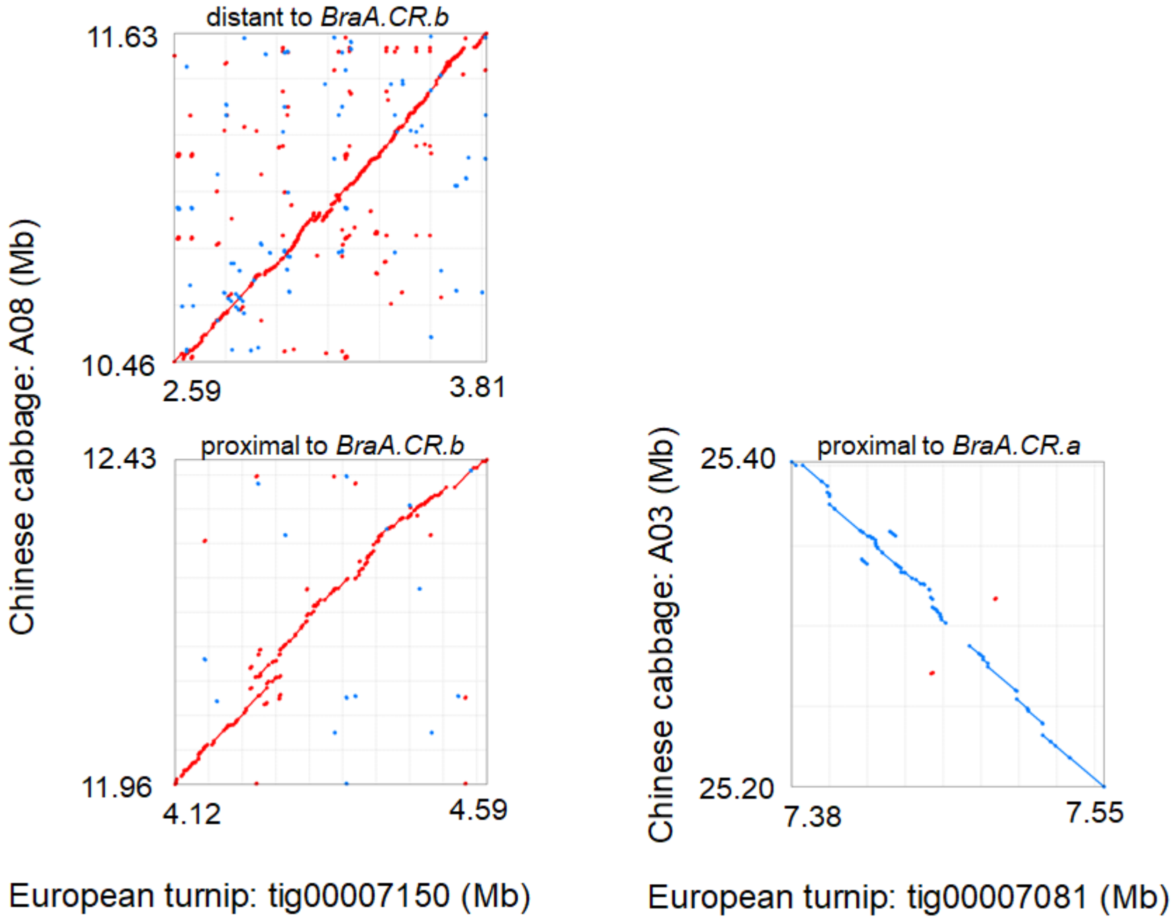

Figure S6. Comparison between genomic regions linked to *BraA.CR.a* and *BraA.CR.b* loci in European turnip and the counterparts in Chinese cabbage.

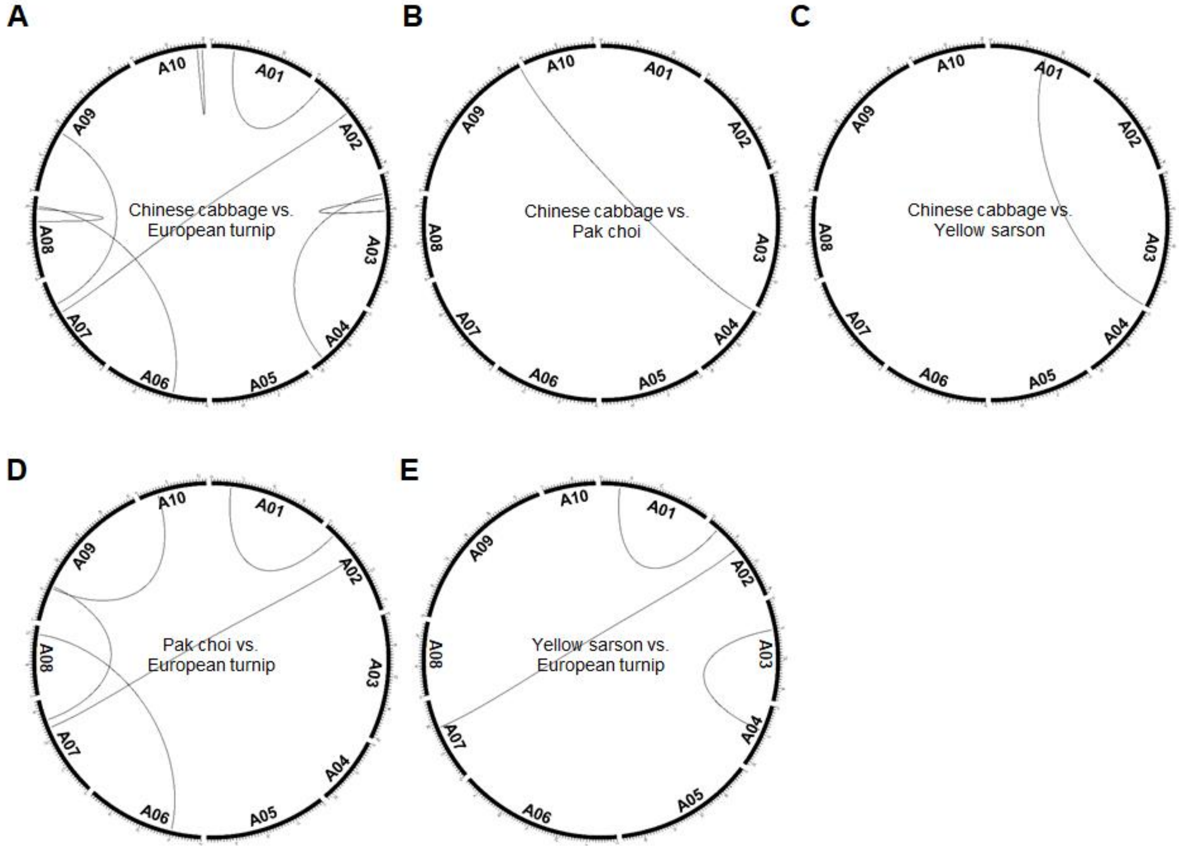

Figure S7. Identification of genome rearrangements between European turnip (ET) and other *B. rapa* subspecies including Chinese cabbage (CC) (v3), pak choi (PC) (Li et al., 2020), and yellow sarson (YS) (Belser et al., 2018). Comparisons: (A) CC vs. ET (presented in Fig. 1B), (B) CC vs. PC, (C) CC vs. YS, (D) PC vs. ET, and (E) YS vs. ET.

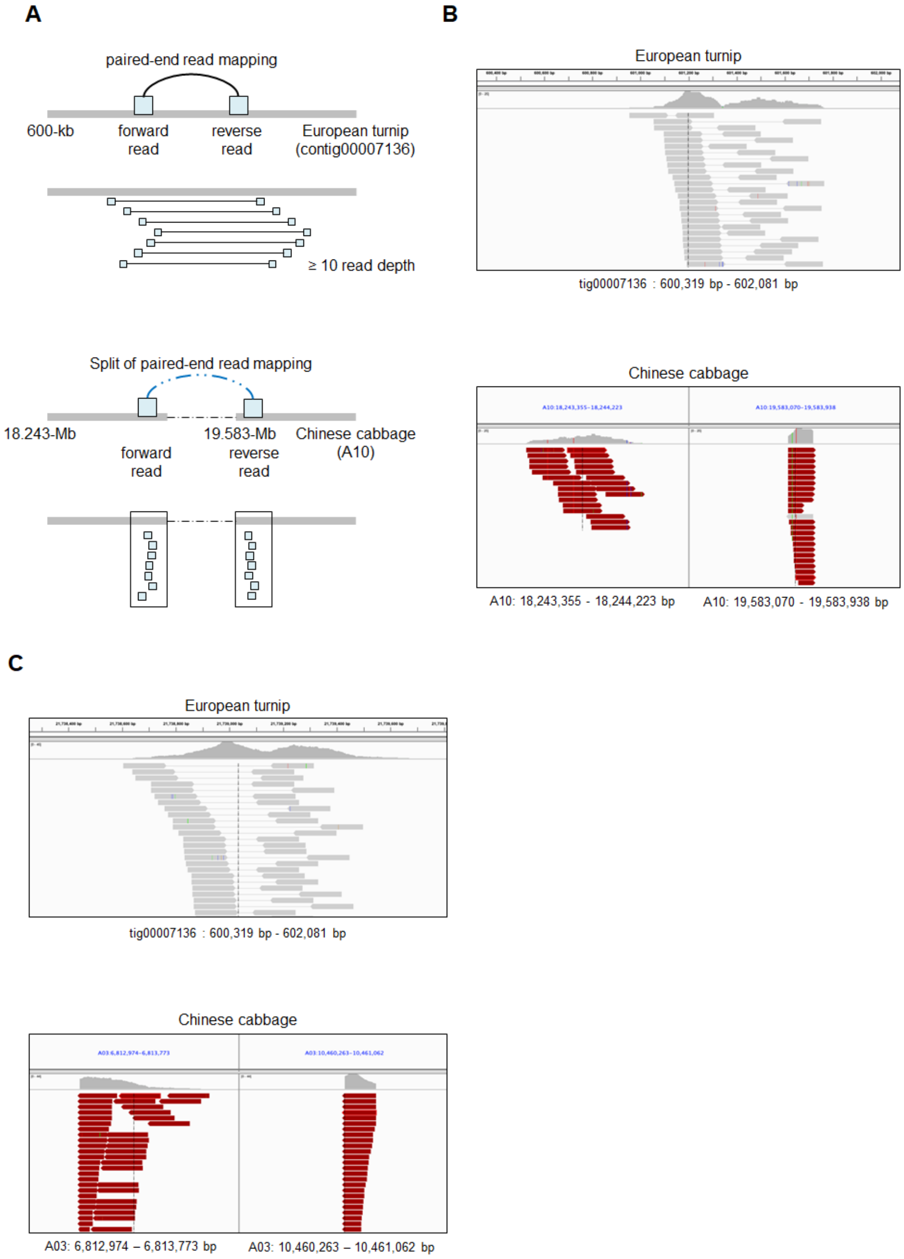

Figure S8. Validation of genome rearrangements between European turnip and Chinese cabbage. (A) The strategy for detecting genome rearrangements based on Illumina paired-end read mapping. For example, paired-end reads of European turnip, which were properly mapped to a 600-kb region in its contig00007136, were mapped to 18.243-Mb region for one side and 19.583-Mb regions for other side in A10 of Chinese cabbage, respectively, showing the collapse of paired-end read mapping in the genome of Chinese cabbage. (B and C) The visualization of read mapping in a hotspot using IGV (https://software.broadinstitute.org/software/igv/).

A

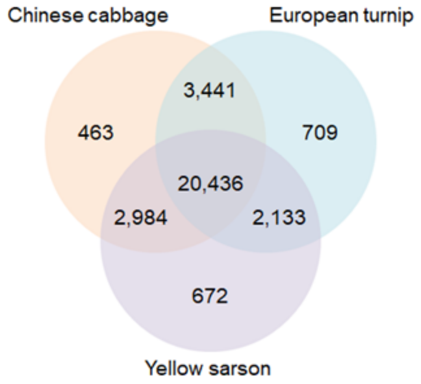

B

InterPro domain	Significance -Log(P-value)		
	CC	ET	YS
Reverse transcriptase zinc-binding domain	6.56	12.25	8.62
Zinc knuckle CX2CX4HX4C		5.92	
Nucleotide-binding, alpha-beta plait		2.70	
RNA recognition motif domain		2.50	
F-box		2.42	
Leucine-rich repeat 2		2.40	
Phosphofructokinase domain		2.15	
Aminotransferase-like, plant mobile domain		2.01	
S locus-related glycoprotein 1 binding pollen coat			21.52
Cytochrome P450			14.27
DVL			8.45
Ribonuclease H-like domain	3.36		7.08
Agenet-like domain			2.83
Tudor-like, plant			2.53
Zinc finger, CCHC-type	3.22		
Peptidase C48, SUMO/Sentrin/Ubl1	2.28		

Figure S9. Orthologous gene clusters among *B. rapa* subspecies sequenced (A) and functional annotation for species-specific genes in three subspecies (B). (A) Orthologous gene clusters among Chinese cabbage (v3), European turnip, and yellow sarson (Belser et al., 2018) were analyzed by using OrthoMCL. (B) Functional protein domains of genes belonging to subspecies-specific orthologous gene cluster were searched using InterProscan. Enrichment of InterPro domains was analyzed by using DAVID with the cutoff of EASE score < 1*E*-02.

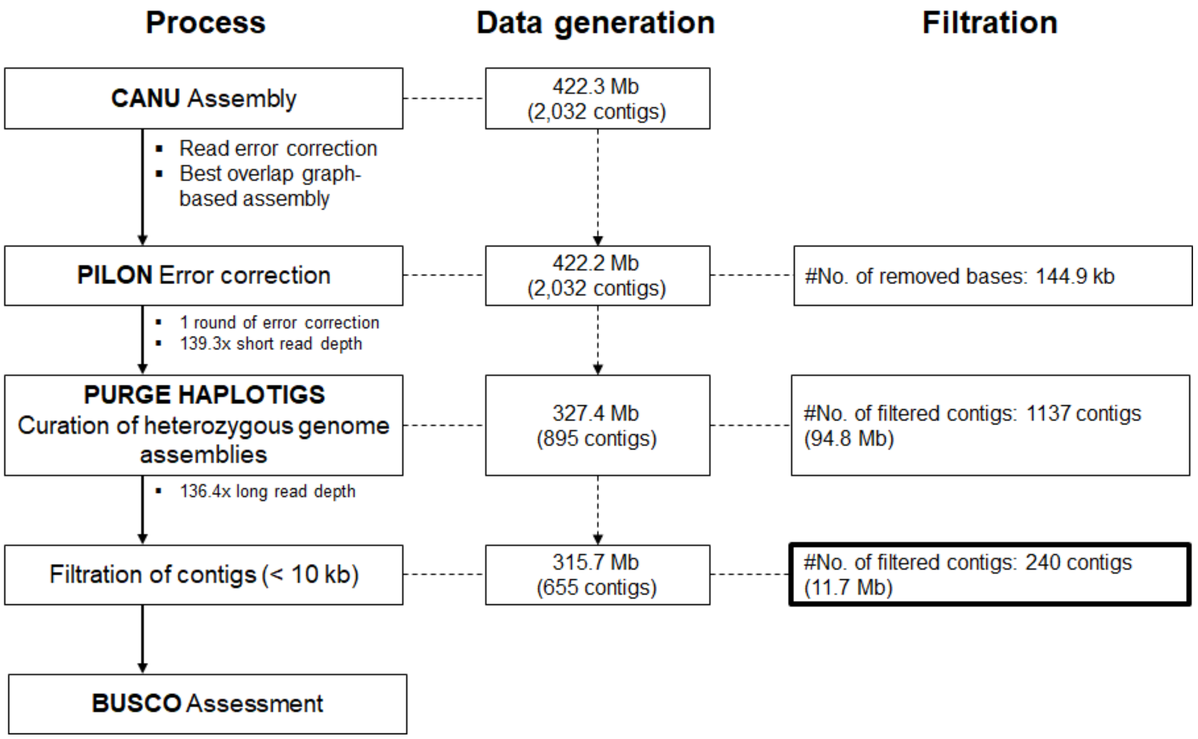

Figure S10. Procedure of genome assembly for European turnip. In procedure, the yield of generated and filtrated data is summarized for each of steps.

Table S1. Summary for whole-genome sequencing data of European turnip.

Sequencing (Platform)	Read length (bp)	No. of reads	Total bases (Gb) (Sequencing depth, x)
Long read sequencing (PacBio)	10,578	6,677,347	70.63 (136.4x)
Short-insert paired-end sequencing (Illumina)	100	477,856,020	72.16 (139.3x)

Table S2. Anchoring contigs of European turnip to the genome of Chinese cabbage.

Chromosome of Chinese cabbage	Total length of conserved blocks (Mb)		
	in Chinese cabbage	in European turnip	Coverage (%) in Chinese cabbage
A01	29.60	24.12	81.50%
A02	31.44	27.30	86.84%
A03	38.15	36.20	94.87%
A04	21.93	20.73	94.53%
A05	28.49	21.95	77.02%
A06	29.17	23.35	80.07%
A07	28.93	26.31	90.94%
A08	22.98	20.93	91.07%
A09	45.16	41.24	91.32%
A10	20.73	18.35	88.55%
Total	296.58	260.48	87.83%

Table S3. Assessment of the genome assembly of European turnip using Benchmarking Universal Single-Copy Orthologs (BUSCO).

Type	European turnip	Chinese cabbage
Complete BUSCOs	423 (99.6%)	423 (99.6%)
Complete and single-copy BUSCOs	350 (82.4%)	359 (84.5%)
Complete and duplicated BUSCOs	73 (17.2%)	64 (15.1%)
Fragmented BUSCOs	1 (0.2%)	1 (0.2%)
Missing BUSCOs	1 (0.2%)	1 (0.2%)

Table S4. Identification of genome rearrangements between European turnip and Chinese cabbage.

No. of hotspot	Chinese cabbage (CC)			European turnip (ET)			No. of conserved fragment	No. of genes in block	
	Aligned region (kb)			Aligned region (kb)					
	Chr.	Start	End	#Contig	Start	End		CC	ET
1	A02	12,643.5	13,041.7	contig00000190	17.7	434.3	12	73	65
	A07	19,223.8	19,473.9		481.8	719.9	8	53	59
2	A01	6,541.1	6,800.9	contig00000704	1,385.8	1,659.0	8	43	51
	A02	2,493.5	3,814.5		17.9	1,363.2	51	288	257
3	A03	6,833.3	7,011.5	contig00007081	21,756.4	21,912.6	7	39	36
	A03	10,470.3	32,568.3		14.9	21,730.4	738	3,937	4,083
4	A03	5,575.4	9,883.2	contig00007082	7.6	2,433.7	69	786	471
	A04	9,474.5	17,996.1		2,472.3	10,942.3	305	1,302	1,376
5	A10	18,057.7	18,217.2	contig00007136	629.5	774.9	7	40	40
	A10	18,966.7	19,568.7		11.5	579.4	21	149	147
6	A06	9,650.1	9,822.0	contig00007935	81.3	290.0	9	22	32
	A08	20,065.6	20,322.4		315.3	582.6	11	66	63
7	A08	15,275.0	15,916.9	contig00008006	17.0	584.1	20	110	113
	A08	19,265.7	19,459.0		603.5	770.7	8	39	37
8	A07	21,403.1	21,916.8	contig00133748	15.5	586.9	20	91	117
	A09	12,363.9	17,725.1		637.0	5,822.9	153	695	653

Table S5. Abundance of transposable elements (TEs) identified in the genome of European turnip.

TE	Searched length (Mb)	%
**Class I elements**	74,403,737	(66.07)
LTR (subtotal)	55,743,062	(49.50)
Ty3/Gypsy	33,187,081	29.47
Ty1/Copia	21,075,761	18.71
Cassandra	534,532	0.47
Caulimovirus	110,190	0.10
ERVK	104,868	0.09
Pao	25,523	0.02
Others/Unknown	705,107	0.63
Non-LTR (subtotal)	18,660,675	(16.57)
LINE	16,252,136	14.43
SINE	2,408,539	2.14
**Class II elements**	38,210,745	(33.93)
DNA (subtotal)	38,210,745	(33.93)
hAT family (with Ac/Charlie/Tag1/Tip100)	13,158,681	11.68
MULE-MuDR	6,365,507	5.65
CMC-EnSpm	5,138,318	4.56
PIF-Harbinger	4,752,496	4.22
Helitron	4,547,650	4.04
TcMar family (with ariner/Pogo/Stowaway/Tc1)	2,748,934	2.44
Others/Unknown	1,499,159	1.33

Table S6. Summary of RNA-Seq of different tissues of European turnip.

Samples	Raw reads	Clean reads (%)	Mapped reads (%)	Uniquely mapped reads (%)	Spliced reads (%)
ECD4 inner leaf_1	53,575,142	52,580,078 (98.14)	47,327,902 (88.34)	45,613,520 (85.14)	18,465,410 (34.47)
ECD4 inner leaf_2	60,300,348	59,523,582 (98.71)	54,193,196 (89.87)	52,210,572 (86.58)	20,646,224 (34.24)
ECD4 outer leaf_1	54,416,502	52,970,948 (97.34)	46,230,010 (84.96)	44,583,200 (81.93)	16,679,664 (30.65)
ECD4 outer leaf_2	63,194,702	61,958,158 (98.04)	55,507,212 (87.84)	53,553,276 (84.74)	19,928,777 (31.54)
ECD4 outer leaf_3	57,775,686	55,980,324 (96.89)	48,593,302 (84.11)	46,930,456 (81.23)	17,995,378 (31.15)
ECD4 main root_1	55,676,998	53,707,262 (96.46)	45,907,882 (82.45)	44,208,654 (79.40)	15,232,827 (27.36)
ECD4 main root_2	56,979,834	55,943,520 (98.18)	49,945,806 (87.66)	48,163,920 (84.53)	16,709,783 (29.33)
ECD4 main root_3	58,162,354	56,707,134 (97.50)	49,476,864 (85.07)	47,692,428 (82.00)	16,172,743 (27.81)
ECD4 lateral root_1	60,672,526	58,771,946 (96.87)	49,381,450 (81.39)	47,685,294 (78.59)	15,795,713 (26.03)
ECD4 lateral root_3	74,434,806	72,847,398 (97.87)	63,282,286 (85.02)	61,160,122 (82.17)	19,825,123 (26.63)

Table S7. Identification of plant resistance *R* genes in the genome of European turnip.

Domains	ET	CC	YS	Bo	Bi	Rs	At
NBS-encoding R genes							
CNL	23	28	26	9	40	24	39
CN	8	3	8	3	8	11	1
TNL	67	78	74	45	112	54	74
TN	14	22	21	22	29	15	15
NL	51	53	29	33	58	69	21
NBS	15	8	13	31	24	16	5
TX	57	55	42	89	77	44	34
Others	17	17	11	8	24	21	15
Membrane-associated R genes							
RLP	119	109	120	75	177	160	71
RLK	742	629	731	559	777	755	519
TM-CC	274	238	223	207	251	221	141

ET, European Turnip; CC, Chinese cabbage; YS, Yellow sarson, Bo, *B. oleracea*; Bi, *B. nigra*; Rs, *R. sativus*; At, *A. thaliana*

Table S8. Mapping of microsatellite markers linked to clubroot resistance loci to the draft assembly of European turnip.

Microsatellite marker primer[1]	Linked marker	Alignment of primer sequence to the assembly of ECD4					Alignment of primer sequence to the genome of Chinese cabbage				
		#ECD4 contig	Forward primer		Reverse primer		Chinese cabbage chromosome	Forward primer		Reverse primer	
			Start (bp)	End (bp)	Start (bp)	End (bp)		Start (bp)	End (bp)	Start (bp)	End (bp)
SASS45c	proximal to *BraA.CR.a*	tig00007081	7,389,147	7,389,167	7,389,455	7,389,475	A03	25,405,695	25,405,715	25,405,391	25,405,411
SASS45b		tig00007081	7,389,208	7,389,228	7,389,432	7,389,451	A03	25,405,634	25,405,654	25,405,415	25,405,434
SASS79b		tig00007081	7,553,845	7,553,864	7,554,063	7,554,082	A03	25,203,690	25,203,709	25,203,402	25,203,421
S33R33	distant to *BraA.CR.b*	tig00007150	2,598,625	2,598,642	2,598,359	2,598,377	A08	10,467,070	10,467,087	10,466,804	10,466,822
S30R30		tig00007150	2,983,800	2,983,814	2,983,529	2,983,547	A08	10,800,075	10,800,093	10,799,804	10,799,822
S23R23		tig00007150	3,534,116	3,534,135	3,533,823	3,533,841	A08	11,348,314	11,348,333	11,348,027	11,348,045
S17R17		tig00007150	3,816,659	3,816,678	3,816,375	3,816,393	A08	11,625,330	11,625,349	11,625,042	11,625,060
S14R14	proximal to *BraA.CR.b*	tig00007150	4,125,571	4,125,589	4,125,281	4,125,299	A08	11,962,215	11,962,233	11,961,933	11,961,951
S11R11		tig00007150	4,359,930	4,359,946	4,359,625	4,359,643	A08	12,241,864	12,241,883	12,241,559	12,241,577
S08R08		tig00007150	4,544,186	4,544,205	4,543,910	4,543,928	A08	12,395,582	12,395,601	12,395,306	12,395,324
S07R07		tig00007150	4,555,737	4,555,755	4,555,460	4,555,478	A08	12,407,122	12,407,140	12,406,841	12,406,859
S06R06		tig00007150	4,590,418	4,590,436	4,590,143	4,590,161	A08	12,433,875	12,433,893	12,433,600	12,433,618

[1] Microsatellite markers reported by Hirani et al. (2018).

[2] (Con)tig00007081 and tig00007150 contain genomic regions spanning clubroot resistance loci *BraA.CR.a* and *BraA.CR.b*, respectively. Supplementary Figure 6 shows the comparison between tig00007081 and its counterpart in A03 (25.2 Mb ~ 25.4 Mb) and between tig00007150 and its counterpart in A08 (10.4 Mb ~ 11.6 Mb and 11.9 Mb ~ 12.4 Mb) of Chinese cabbage.

[3] Hirani, A. H., Gao, F., Liu, J., Fu, G., Wu, C., McVetty, P., Duncan, R. W., and Li, G. (2018). Combinations of Independent Dominant Loci Conferring Clubroot Resistance in All Four Turnip Accessions (*Brassica rapa*) From the European Clubroot Differential Set. *Frontiers in plant science*, 9, 1628. https://doi.org/10.3389/fpls.2018.01628.

Table S9. List of *R* genes within conserved blocks that are linked to clubroot resistance loci.

Linked to *CRa/b* marker	Plant resistance *R* genes											
	Chinese cabbage					Collinearity of genes between Chinese cabbage and ECD4	ECD4					
	Genomic regions (bp)	Gene ID	Position (bp)		Domain		ECD4 Assembly (counterpart)	Gene ID	Position (bp)		Domain	Note
			Start	End					Start	End		
Proximal to *BraA.CR.a*	A03: 25,203,402 - 25,405,715	BraA03g049600.3C	25,213,983	25,217,814	RLK	Yes: BraA03g049600.3C - GENE22552.1	tig00007081	GENE22552.1	7,488,865	7,491,493	RLK	
Distant to *BraA.CR.b*	A08: 10,466,804 - 11,625,349					No	tig00007150	GENE35902.1	3,430,277	3,433,000	RLP	
		BraA08g012910.3C	11,232,393	11,235,117	RLP	Yes: BraA08g012910.3C - GENE35904.1		GENE35904.1	3,445,195	3,447,572	RLP	
		BraA08g012940.3C	11,267,185	11,268,651	RLK	Yes: BraA08g012940.3C - GENE35906.1		GENE35906.1	3,456,500	3,457,966	RLK	
		BraA08g012990.3C	11,291,257	11,292,552	RLP	Yes: BraA08g012990.3C - GENE35908.1		GENE35908.1	3,473,295	3,475,298	RLP	
						No		GENE35916.1	3,524,122	3,524,888	TM-CC	
						No		GENE35952.1	3,730,220	3,739,922	TNL	*Rcr6* homolog[1]
						No		GENE35955.1	3,752,622	3,757,359	TNL	*Rcr6* homolog
Proximal to *BraA.CR.b*	A08: 11,961,933 - 12,433,893	BraA08g014350.3C	12,162,113	12,169,372	RLK	Yes: BraA08g014350.3C - GENE36062.1	tig00007150	GENE36062.1	4,290,699	4,296,694	RLK	
						No		GENE36077.1	4,366,191	4,367,740	TM-CC	
		BraA08g014480.3C	12,271,553	12,276,276	TNL	Yes: BraA08g014480.3C - GENE36082.1		GENE36082.1	4,399,833	4,403,361	NL	*Rcr6* homolog
		BraA08g014530.3C	12,326,397	12,329,524	RLK	Yes: BraA08g014530.3C - GENE36087.1		GENE36087.1	4,433,805	4,436,979	RLK	

[1] Homology to *Rcr6* [*]: GENE35952.1 (BLASTP *E*-value: 4*E*-63), GENE35955.1 (3*E*-24), GENE36082.1 (0.0)

[*] *Rcr6*: Chang, A., Lamara, M., Wei, Y. *et al.* (2019). Clubroot resistance gene *Rcr6* in *Brassica nigra* resides in a genomic region homologous to chromosome A08 in *B. rapa*. BMC Plant Biol. 19, 224. doi:10.1186/s12870-019-1844-5

Table S10. BUSCO scores with multiple rounds of error correction in Pilon.

Type	1st round	2nd round	3rd round	4th round
Complete BUSCOs	423 (99.6%)	421 (99.0%)	421 (99.0%)	421 (99.0%)
Complete and single-copy BUSCOs	350 (82.4%)	344 (80.9%)	344 (80.9%)	344 (80.9%)
Complete and duplicated BUSCOs	73 (17.2%)	77 (18.1%)	77 (18.1%)	77 (18.1%)
Fragmented BUSCOs	1 (0.2%)	2 (0.5%)	2 (0.5%)	2 (0.5%)
Missing BUSCOs	1 (0.2%)	2 (0.5%)	2 (0.5%)	2 (0.5%)
